# Aerobic Cultivation of *Mucor* Species Enables the Deacidification of Yogurt Acid Whey and the Production of Fungal Oil

**DOI:** 10.3390/foods12091784

**Published:** 2023-04-25

**Authors:** Xingrui Fan, Viviana K. Rivera Flores, Timothy A. DeMarsh, Dana L. deRiancho, Samuel D. Alcaine

**Affiliations:** Department of Food Science, Cornell University, Ithaca, NY 14850, USA; xf75@cornell.edu (X.F.); vkr6@cornell.edu (V.K.R.F.);

**Keywords:** *Mucor circinelloides*, *Mucor genevensis*, acid whey, biomass, lactic acid, biodiesel, fungal oil, γ-linolenic acid

## Abstract

As the Greek-style yogurt market continues to experience prosperous growth, finding the most appropriate destination for yogurt acid whey (YAW) is still a challenge for Greek yogurt manufacturers. This study provides a direct alternative treatment of YAW by leveraging the abilities of *Mucor circinelloides* and *Mucor genevensis* to raise the pH of YAW and to produce fungal biomass with a high lipid content. Aerobic cultivations of these species were conducted in YAW, both with and without the addition of lactase, at 30 °C, and 200 rpm agitation. The density, pH, biochemical oxygen demand (BOD), biomass production, lipid content, fatty acid profile, and sugar and lactic acid concentrations were regularly measured throughout the 14-day cultivations. The data showed that *M. genevensis* was superior at deacidifying YAW to a pH above 6.0—the legal limit for disposing of cultured dairy waste. On the other hand, *M. circinelloides* generated more fungal biomass, containing up to 30% *w*/*w* of lipid with high proportions of oleic acid and γ-linolenic acid. Additionally, the treatments with lactase addition showed a significant decrease in the BOD. In conclusion, our results present a viable treatment to increase the pH of YAW and decrease its BOD, meanwhile generating fungal oils that can be further transformed into biodiesel or processed into functional foods or dietary supplements.

## 1. Introduction

In the past two decades, Greek yogurt has gained popularity and now occupies more than 50% of the U.S. yogurt market share due to its nutritional profile and potential health benefits [1]. During its manufacturing processes, yogurt acid whey (YAW) is separated as a by-product following the acid coagulation step in order to provide the Greek yogurt with a creamier and thicker mouthfeel. However, YAW cannot be disposed of directly due to environmental concerns: the dissolved oxygen in the water stream can be diminished as the microorganisms proliferate, given the abundance of nutrients in YAW [2,3]. This later results in severe damage to the aquatic environment, such as algae bloom and death of aquatic lives [2,3]. This potential hazard of disposing of YAW can be attributed to its high biochemical oxygen demand (BOD) and chemical oxygen demand [2,4]. The Code of Federal Regulations (CFR) Title 40 Part 405 specified the limitations on pH and BOD of cultured dairy effluents, and YAW does not meet these requirements [2,5]. Two common usages of YAW are to apply this effluent onto farmlands and to mix it into animal feed [6]. However, land applications of YAW can lead to a fluctuation of soil pH, which can negatively affect the growth of crops [6,7], and excessive intake of YAW by animals can result in digestive problems [6].

In recent years, scientists have conducted research investigating the conversion of YAW into food ingredients or other resources through various means. For instance, fermentations of YAW have been researched as potential strategies to produce bioderived resources such as ethanol, galactose, lactobionic acid, galactooligosaccharides, and medium-chain carboxylic acids [8,9,10,11,12]. Processing unit operations, including membrane filtrations, have also been studied to convert YAW into whey proteins, milk minerals, and monosaccharide products [6,13]. The incorporation of YAW into food formulations has also been explored as a potential usage of this waste stream [14,15]. Though these promising applications could partially alleviate the concern over YAW disposal, an easy and cost-effective processing method for YAW is still in demand due to two considerations: the Greek yogurt market is projected to exceed USD 13 billion in the next four years [16], and a tremendous quantity of YAW is produced by the Greek yogurt industry—with respect to weight, YAW is produced at double to triple the amount of the actual Greek yogurt produced [3].

*Mucor* is a fungal genus commonly regarded as a food contaminant. Marcus et al. [17] observed that *Mucor genevensis* slightly increased the pH of whey permeate when it was cultivated in aerobic conditions. This finding provided a promising strategy to deacidify YAW by utilizing the metabolism of *M. genevensis*, leading to a potential treatment for YAW prior to disposal or other processing methods [17]. Chan et al. [18] explored the use of *Mucor circinelloides* in lactose-hydrolyzed cheese whey permeate to produce a biomass containing more than 20% *w*/*w* lipid. Such fungal oil is also known as single cell oil and can be utilized in the liquid fuel production chain [19]. It is worth mentioning that the triglycerides produced from *M. circinelloides* are rich in monounsaturated fatty acids (MUFAs) and γ-linolenic acid (GLA), both of which are deemed to have health benefits [20,21]. Given this information, we further investigated the potential of cultivating *M. genevensis* and *M. circinelloides* in YAW to generate fungal oils, in addition to deacidifying the waste stream.

The objectives of this study are to explore a deacidification method for YAW by cultivating *M. genevensis* and *M. circinelloides* in aerobic conditions and to compare the amounts and fatty acid compositions of the fungal oils produced by these two species. The outcomes of this study can offer the Greek yogurt industry a simple treatment of YAW, and it may benefit manufacturers by offering savings on processing and transportation costs while also producing a potentially profitable oil-rich fungal biomass.

## 2. Materials and Methods

### 2.1. Experimental Design

The cultivations of *Mucor* species were performed in 100 mL of YAW at 30 °C with an agitation of 200 rpm. Since previous studies reported that *M. genevensis* and *M. circinelloides* cannot metabolize lactose [17,22], the effect of lactose hydrolysis in YAW on the performances of both species was also evaluated. Therefore, four treatments were set up: (i) *M. circinelloides* in lactose-hydrolyzed YAW (MC+E); (ii) *M. circinelloides* in non-hydrolyzed YAW (MCNH); (iii) *M. genevensis* in lactose-hydrolyzed YAW (MG+E); and (iv) *M. genevensis* in non-hydrolyzed YAW (MGNH). Accordingly, the inoculated *Mucor* species and the usage of lactase were designed to be the two independent, categorical variables in this experiment. After these 100 mL scale cultivations, another set of cultivations was conducted in 620 mL YAW, with the same four treatments, in order to obtain sufficient samples for fatty acid profiling and BOD analysis.

### 2.2. Microorganisms and Preparation of the Culture Substrates

*M. genevensis* (FSL ARGTD-0021) was previously isolated and identified from a commercial dairy product. *M. circinelloides* (FSL ARGTD-0044) was isolated and identified from bokashi. For both species, the isolates were cryopreserved at −80 °C. YAW was obtained from local Greek yogurt manufacturers and was sterilized in an autoclave at 121 °C for 20 min. The hydrolysis of lactose in YAW was performed by using a commercial lactase product (Maxilact A4, Royal DSM, Delft, The Netherlands), which had been sterilized using a 0.2 μm pore-sized cellulose acetate syringe filter (Corning Inc., Corning, NY, USA). The enzyme was added 24 h prior to the inoculation of the *Mucor* species, targeting a level of 120 acid lactase activity units (ALUs) per gram of lactose in the YAW for treatments MC+E and MG+E.

### 2.3. Starter Preparation

Both *Mucor* species were resuscitated on potato dextrose agar (Hardy Diagnostics, Santa Maria, CA, USA) with a 25 mg supplement of chloramphenicol (Sigma Life Science, Sigma-Aldrich Co., St. Louis, MO, USA) per liter of medium, which will thusly be referred to as PDA+CAM. Given *Mucor* has dimorphism characteristics and can grow differently depending on the oxygen level [23], the inoculated plates were incubated at 30 °C for 48 h in a GasPak EZ anaerobic pouch system (Becton, Dickinson and Company, Sparks, MD, USA) to ensure that the microbes remained in their yeast forms, thus avoiding spreading growth and allowing for the enumeration of cells under a microscope. Then, for each species, an isolated colony was transferred into a culture tube containing 10 mL of a 120 g/L dry malt extract (DME, Briess Malt & Ingredients Co., Chilton, WI, USA) solution, which had been sterilized using a 0.45 μm polyethersulfone membrane filter (VWR International, Radnor, PA, USA). These tubes were incubated at 30 °C for 24 h in a GasPak EZ anaerobic chamber system (Becton, Dickinson, and Company). After this incubation period, the contents of each tube were transferred to a separate 250 mL Erlenmeyer flask containing 220 mL of filter-sterilized 120 g/L DME solution. These flasks were then incubated anaerobically at 30 °C for 72 h and then used as the starters for *Mucor* cultivations.

### 2.4. Inoculation and Biomass Cultivation

Following the incubation of the starters, the concentration of cells in each culture was estimated by counting under a microscope with a hemocytometer. Based on the estimated cell concentration, the volume of culture for each treatment was calculated to achieve an inoculation rate of 5 × 10^4^ cells/mL in 800 mL of YAW. This volume of starter was transferred into a 250 mL conical tube and centrifuged at 2602× *g* for 10 min to precipitate the cells. The supernatant was discarded, and the cells were resuspended in 100 mL of YAW and then transferred into a one-liter Scott bottle containing 700 mL YAW. The inoculated YAW was then vortexed and separated into eight 250 mL flasks, each containing 100 mL of the inoculated YAW. The flasks were incubated at 30 °C with an agitation of 200 rpm.

### 2.5. Sampling and Analysis Methods

In each treatment, eight flasks of the same culture were prepared: one flask was used to check the pH every day during the incubation period, while the remaining seven were used for sampling every two days and for further analyses. More specifically, on each of the even-numbered days during the two-week cultivation, one flask among the reserve of seven in each treatment was removed from the incubator and was used to measure and record the pH, density, microbial plate count, and fungal biomass.

For each sample flask, a 0.5 mL sample was taken for microbial counts and was serially diluted with phosphate-buffered saline. Dilutions were then plated on PDA+CAM, followed by a 48 h incubation period at 30 °C in an anaerobic chamber. The biomass was then separated from the remaining spent medium by filtration with a piece of pre-weighed Grade 90 (44 × 36 weave) cheesecloth. The biomass, together with the piece of cheesecloth, was dried in a commercial food dehydrator (Commercial Chef, New York, NY, USA) at 60 °C until a constant weight was obtained, and the biomass with the cheesecloth was then cooled and stored in a desiccator for further analysis.

For the spent medium that passed through the cheesecloth, pH was measured using a Halo2 Milk pH Tester (Hanna Instruments, Smithfield, RI, USA). The rest of the spent medium was autoclaved at 121 °C for 20 min in order to prevent the potential spread of mold spores during further analyses. Following sterilization, the density was measured by a DMA 35 Densitometer (Anton Paar, Graz, Austria).

Samples of the autoclaved spent media from days 6 and 14, along with the uninoculated YAW, were analyzed for sugar and lactic acid concentrations using enzymatic kits. Lactose and galactose concentrations were determined using K-LACGAR for lactose and D-galactose (Megazyme, Wicklow, Ireland). Samples were also sent to Cornell AgriTech Craft Beverage Analytical Lab (Geneva, NY, USA) for glucose and lactic acid analysis; concentrations were determined using Food Quality Enzymatic Analysis kits (BioSystems S.A., Barcelona, Spain).

The dried biomass was weighed after cooling to room temperature in a desiccator. Biomass samples collected on days 2, 8, and 14 were analyzed for lipid content using the acid hydrolysis Mojonnier ether extraction method [24].

### 2.6. Determinations of Fatty Acid Profile in Fungal Lipids and Biochemical Oxygen Demand in Spent Cultivation Media

To further investigate the nutritional value and potential uses of fungal lipids, another set of fermentations was conducted to collect enough biomass for fatty acid profile analysis. For each biological replicate, the four treatments indicated in Section 2.2 were applied. In each treatment, two 1 L flasks, each containing 620 mL of culture, were prepared: one was sampled on day 10, as a sample from that day had resulted in the maximum biomass yield from the fastest growing treatment in the 100 mL scale fermentations; the other was sampled on day 14.

*Mucor* starters were freshly prepared for each replicate using the same procedures mentioned in Section 2.3. Harvesting and drying of fungal biomass was performed using the same procedures described in Section 2.5, and the dried biomass was shipped to Eurofins Food Chemistry Testing Madison, Inc. (Madison, WI, USA) for fatty acid analysis (FALC_S).

The spent media were autoclaved as described in Section 2.5 and then sent to Certified Environmental Services, Inc. (North Syracuse, NY, USA), along with the uninoculated YAW, for the 5-day BOD test.

### 2.7. Statistical Analyses

All the statistical analyses in this study were performed using JMP Pro 17.0.0 (SAS Institute, Cary, NC, USA). The statistically significant level was set to 0.05 for all of the analyses. For each sampling timepoint, comparisons amongst all four treatments were performed using a one-way ANOVA to examine whether there was a significant difference amongst the data from these four treatments. When a significant difference was found, a Tukey’s HSD test was applied to determine which treatment or treatments were significantly different from others. In addition, a two-way ANOVA was applied based on the two independent variables—the inoculated species and the usage of lactase—in order to determine whether these two variables, and the interaction between them, contributed to the significant difference found among the four treatments. Paired *t*-tests were conducted on the concentrations of lactose across different timepoints in order to investigate whether *Mucor* species can metabolize lactose or not.

## 3. Results and Discussion

### 3.1. Utilizations of Lactose, Galactose, and Glucose by Mucor Species

**Figure 1:**
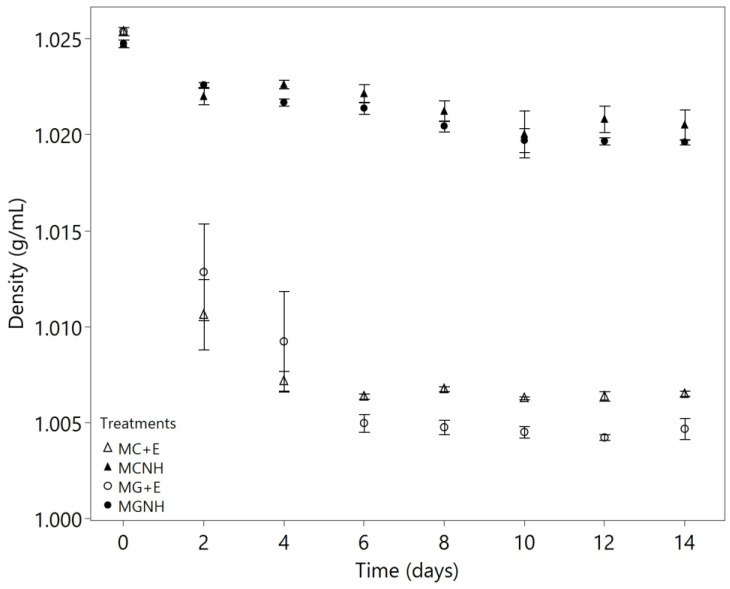
Densities (g/mL) of *Mucor* cultures during 14-day aerobic cultivation at 30 °C and 200 rpm agitation in 100 mL yogurt acid whey. The markers show the means of biological triplicates, and the error bars refer to one standard error from the mean. MC = *M. circinelloides*; MG = *M. genevensis*; +E = with lactase addition; NH = without lactase addition.

The uninoculated YAW on average contained 39.11 g/L lactose, 6.72 g/L galactose, and 0.18 g/L glucose. In the treatments where lactose had been hydrolyzed into glucose and galactose, both *M. genevensis* (MG+E) and *M. circinelloides* (MC+E) were able to consume these monosaccharides to concentrations below 0.1 g/L by day 6 (Table 1). Previous studies on *M. circinelloides* [22] and *M. genevensis* [25] also showed their ability to metabolize galactose and glucose. The rapid consumption of sugars by both species was also reflected in the densities of the lactose-hydrolyzed YAW spent media: the densities dropped from 1.0254 g/mL to 1.0064 and 1.0050 g/mL for MC+E and MG+E, respectively (Figure 1).

For YAW without the addition of lactase, both *M. circinelloides* (MCNH) and *M. genevensis* (MGNH) nearly depleted the 6.72 g/L of galactose within six days (Table 2). In contrast, the lactose concentrations in these cultures did not change significantly during the two-week cultivation period based on the results of the paired *t*-tests. When the corresponding lactose concentrations were compared with those on day 0 for MCNH, *p* = 0.1898 (day 6) and *p* = 0.1479 (day 14). Similarly, for MGNH, *p* = 0.8004 (day 6) and *p* = 0.1641 (day 14). This result suggested that both *M. genevensis* and *M. circinelloides* cannot utilize lactose, which matched the findings from previous studies [17,22]. As both species were incapable of consuming lactose, the densities of cultures without the addition of lactase did not decrease as much as those that had been lactose-hydrolyzed (Figure 1). The previous study conducted by Marcus et al. [17] in whey permeate demonstrated that the density remained almost unchanged throughout a 35-day aerobic cultivation of *M. genevensis*. In the present study, the consumption of galactose, lactic acid, and other nutrients was considered to be responsible for the slight decreases in densities in YAW, as these constituents were taken up by both species. Cultivations of other fungal species that have lactase activities, such as *Mucor pusillus* and *Mucor miehei* [26,27], can be further explored in YAW to assess their performances on the deacidification and fungal oil production.

**Figure 2:**
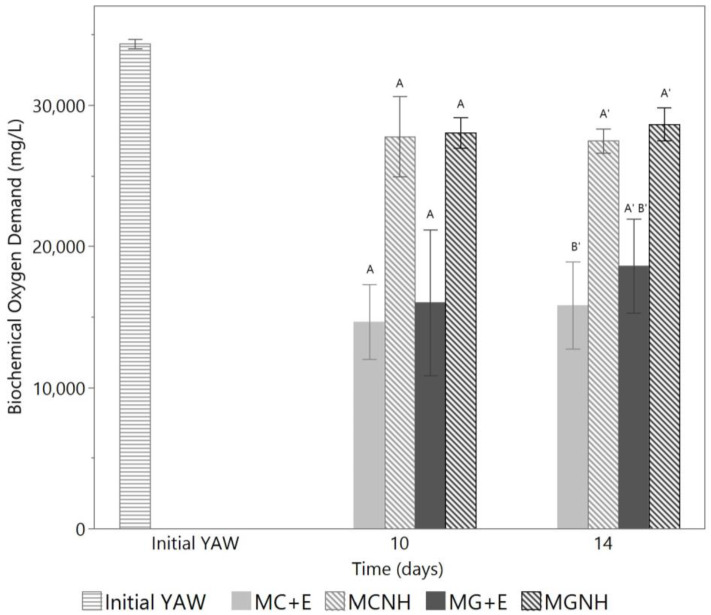
Changes in biochemical oxygen demand during 14-day aerobic cultivations of *Mucor* species at 30 °C and 200 rpm agitation in 620 mL yogurt acid whey (YAW). The bars show the means of biological triplicates, and the error bars refer to one standard error from the mean. On each day, the bars denoted with different letters are significantly different from each other. MC = *M. circinelloides*; MG = *M. genevensis*; +E = with lactase addition; NH = without lactase addition.

As a result of the rapid consumption of sugars in lactose-hydrolyzed YAW, its BOD was significantly reduced (Figure 2), since sugars account for roughly 2/3 of the total solids in YAW [2]. By comparison, in cultures that were not lactose-hydrolyzed, BODs were found to be much higher, as neither *Mucor* species were able to consume lactose. However, it is important to acknowledge that the spent media, even those in which the lactose had been hydrolyzed and then consumed by *Mucor* species, still had BOD values higher than the standards allowed for disposal [5]. This could be ascribed to the fungal biomass left behind in the spent media. To address this problem, further processing methods, such as centrifugation or biomass pelletization, may help to remove the additional biomass [28]. It is worth noticing that even if the BOD level was reduced below the limitation specified by 40 CFR 405, a proper removal or inactivation method of residual *Mucor* cells and spores, such as using microfiltration or thermal treatment, would still be needed. This is because the release of spent media with metabolically active *Mucor* cells into water streams may induce pollution or infectious diseases in aquatic lives caused by their overgrowth [29]. If the BOD remains high after the removal of *Mucor* biomass, further processing methods such as spray drying could also be more easily applied to the spent media, which provides a solution to this high BOD residue to fully recover the nutrients in YAW [30].

### 3.2. Deacidification of Yogurt Acid Whey

**Figure 3:**
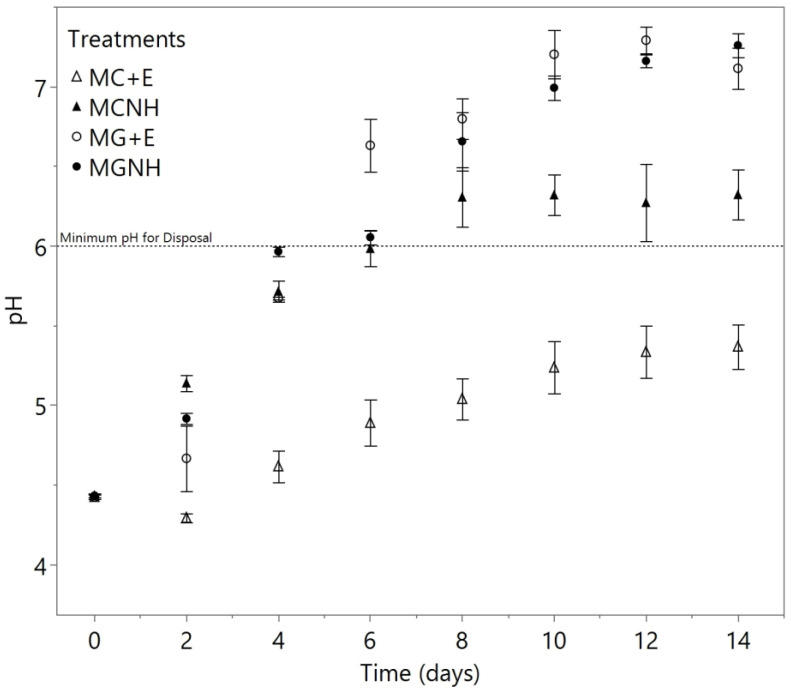
The changes in pH during the 14-day aerobic cultivations of *Mucor* species at 30 °C and 200 rpm agitation in 100 mL yogurt acid whey. The markers show the means of biological triplicates, except for MCNH on day 12 where an outlier was removed. The error bars refer to one standard error from the mean. MC = *M. circinelloides*; MG = *M. genevensis*; +E = with lactase addition; NH = without lactase addition.

During the two-week aerobic incubation period, the pH of all cultures increased. Both MG+E and MGNH rose the pH from 4.43 to 7.12 ± 0.13 and 7.26 ± 0.08, respectively (Figure 3). MCNH stabilized at a pH of 6.3 after day 8, while MC+E just reached a pH of 5.37 ± 0.14 on day 14 (Figure 3). Regarding these changes in the pH, at least one treatment was found to be significantly different (*p* = 0.003) from the others on day 2. The pH decreased in MC+E, whereas the other three treatments all displayed pH increases. Regarding the treatments inoculated with *M. genevensis*, we noticed that MGNH resulted in a higher pH on day 4, and then it was surpassed by the pH of MG+E on day 6. We speculate that this phenomenon could be ascribed to a delayed consumption of lactic acid when *M. genevensis* was in an environment with plenty of glucose and galactose, which could preferably be metabolized as carbon sources.

Using a two-way ANOVA, lactase usage (*p* = 0.001) as well as the interaction between inoculation species and lactase usage (*p* = 0.025) were determined to have significant effects on the responses of pH by day 2. A *p*-value less than 0.0001 was found on the effect of the inoculation species starting on day 4 and lasted throughout the remaining days of the cultivation period, suggesting that there was a significant difference between the ability of *M. genevensis* and *M. circinelloides* to raise the pH of YAW cultures. This result indicates that *M. genevensis* would be a better candidate for neutralizing the pH of YAW waste stream. According to 40 CFR 405 [5], the effluent pH for cultured dairy products is limited to the range of 6.0–9.0. For all treatments except MC+E, the final pH of the spent media fell in this range, suggesting that the aerobic cultivation method of *Mucor* may be applied as a pre-disposal deacidification treatment for YAW.

**Figure 4:**
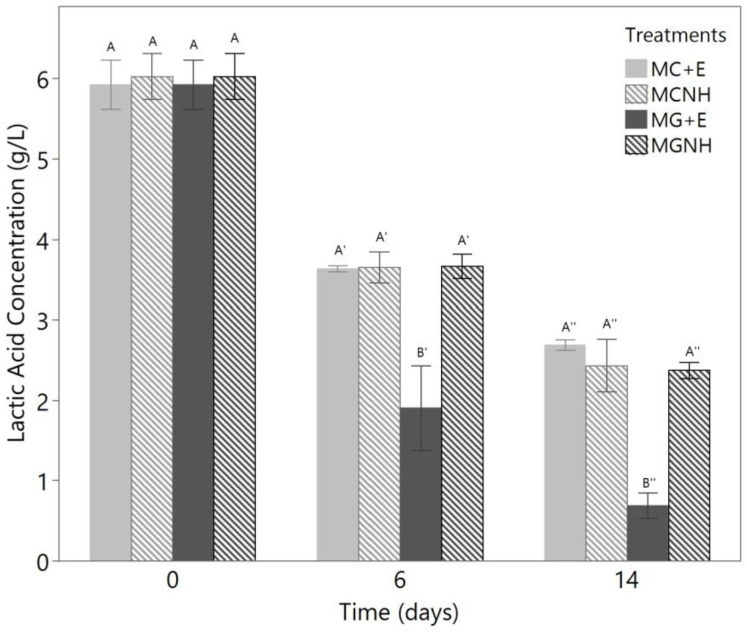
Changes in lactic acid concentrations during the 14-day aerobic cultivation of *Mucor* species in 100 mL yogurt acid whey at 30 °C and 200 rpm agitation. The bars show the means of biological triplicates, and the error bars refer to one standard error from the mean. On each day, the bars denoted with different letters are significantly different from each other. MC = *M. circinelloides*; MG = *M. genevensis*; +E = with lactase addition; NH = without lactase addition.

As mentioned before, a slight increase in pH was found by Marcus et al. when *M. genevensis* was cultivated in whey permeate solution, and this increase in pH was found to be related to a decrease in lactic acid concentration [17]. Botha et al. [22] also identified *M. circinelloides* as a species that was capable of assimilating lactic acid. Therefore, the concentrations of lactic acid in the spent media were tracked and graphed in Figure 4.

All treatments exhibited a decrease in lactic acid concentration throughout the 14-day cultivation period. MG+E decreased the lactic acid concentration to 0.69 ± 0.16 g/L by day 14. There were significant differences among the lactic acid concentrations of the four treatments on both day 6 (*p* = 0.0061) and day 14 (*p* = 0.0003) as determined by one-way ANOVA. For both days, the following Tukey’s tests suggested that MG+E resulted in a significantly lower concentration of lactic acid as compared to the other three treatments. The two-way ANOVA determined that the inoculation species, lactase addition, and the interaction between these two variables were significant to the decreases in the lactic acid concentrations.

Although on both day 6 and day 14 we observed no significant differences in the lactic acid concentrations of MGNH, MC+E, and MCNH, the differences in their pHs were considered significant. Such a discrepancy suggests that, although lactic acid consumption is linked to an increase in pH, it may not be the sole contributor. One hypothesis is that the *Mucor* species behaved differently in their assimilation of citric acid, which exists in the second largest amount among all the organic acids in YAW that had been screened by Menchik et al. [2]. In addition, the buffering capacity of YAW spent media may vary depending on the metabolism of the cultivated species.

The consumption of lactic acid could also allow processors to spray dry the spent medium with the purpose of recovering the nutrients that have not been utilized by *Mucor*. It has been reported that spray drying YAW is a challenging operation as the dried particles will stick to the drying chamber and cyclone [30]. This is caused by the presence of lactic acid, which affects the crystallization of lactose and decreases the glass transition temperature of the particles containing protein and lactose [30]. Since the lactic acid concentration dropped significantly after 14 days of *Mucor* cultivation, the application of the spent media can potentially be explored in future studies in order to recover most of the nutrients in YAW.

### 3.3. Biomass and Fungal Oil Production

**Figure 5:**
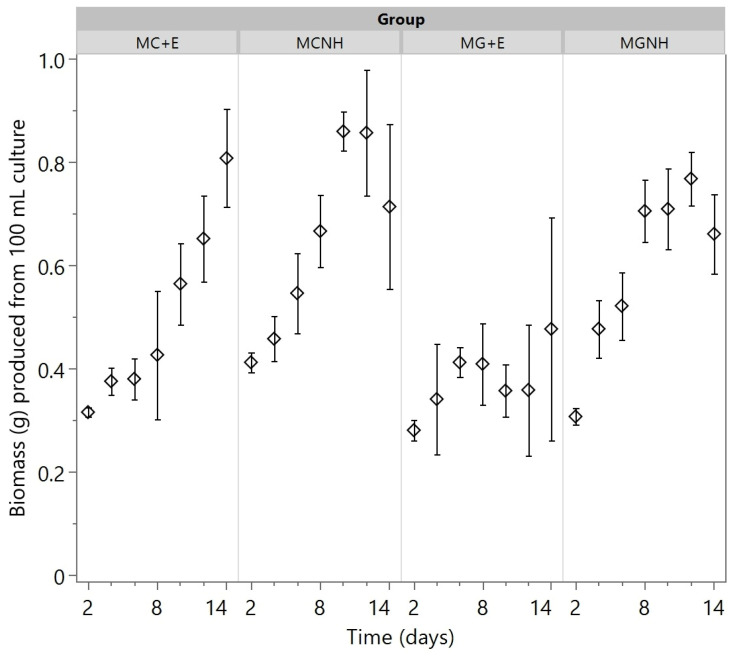
Dried fungal biomass production during the aerobic cultivations of *Mucor* species at 30 °C and 200 rpm agitation in 100 mL yogurt acid whey. The markers show the means of biological triplicates, and the error bars refer to one standard error from the mean. MC = *M. circinelloides*; MG = *M. genevensis*; +E = lactase addition; NH = no lactase addition.

*Mucor* biomass was already produced at day 2, and it gradually accumulated until the end of the 14-day cultivation period in three out of four treatments (MC+E, MCNH, and MGNH). In contrast, the biomass yield of MG+E did not increase after day 6 but it fluctuated around 0.4 g per 100 mL of culture (Figure 5). The one-way ANOVA determined that the amounts of biomass produced in the four treatments had significant differences on day 2 (*p* = 0.0024), day 10 (*p* = 0.0031), and day 12 (*p* = 0.0363). On day 2, significantly more biomass was produced from MCNH in comparison to the other three treatments, and both the inoculation species (*p* = 0.0028) and the lactase usage (*p* = 0.0058) were determined to be significant for this difference. These two factors were also found to have statistical significances on day 10 (*p* = 0.0234 for inoculated species; *p* = 0.0010 for lactase usage). On day 12, however, only the lactase usage was determined to be a statistically significant factor (*p* = 0.0157).

On average, more biomass was produced from YAW without a lactase addition during the first 12 days of cultivation for both species (Figure 5). Nonetheless, on day 14, the amount of biomass produced by MC+E (0.81 ± 0.09 g per 100 mL culture) exceeded that from MCNH (0.71 ± 0.16 g per 100 mL culture). These findings do not match the outcomes from a previous study performed in cheese whey permeate by Chan et al. [18], where *M. circinelloides* was observed to consistently have more biomass produced in the lactose-hydrolyzed substrate. The differences in substrate could be the reason for this discrepancy. In addition, as the cultivation was conducted in flasks covered with aluminum foil to avoid potential spore cross-contaminations, there is a possibility that the exchange of gas was not sufficient. In this case, as oxygen was consumed in the flask through aerobic respiration, some *Mucor* cells might have shifted into the yeast form [23], which is favored in anaerobic conditions, and could not be harvested for the biomass measurement. The maximum *M. circinelloides* biomass yield obtained in this study (0.86 ± 0.037 g/100 mL culture) was comparable to that produced from cheese whey permeate (9.09 ± 0.91 g/1 L culture), where the researchers optimized the fermentation temperature (33.6 °C) and the substrate pH (4.5) for maximum biomass production [18].

**Figure 6:**
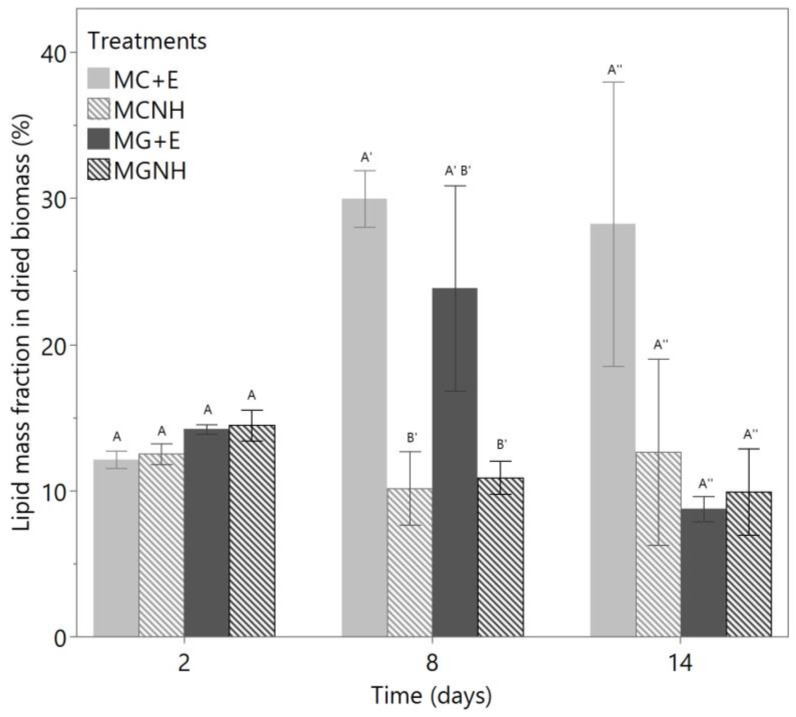
Lipid mass fraction (%) in the dried *Mucor* biomass produced from the aerobic cultivations at 30 °C and 200 rpm agitation in 100 mL yogurt acid whey. The bars show the means of biological triplicates, and the error bars refer to one standard error from the mean. On each day, the bars denoted with different letters are significantly different from each other. MC = *M. circinelloides*; MG = *M. genevensis*; +E = with lactase addition; NH = without lactase addition.

The lipid mass fraction in dried *Mucor* biomass is presented in Figure 6. On day 2, no significant difference in the lipid percentages was found among the treatments (*p* = 0.115). On day 8, the differences among treatments were significant (*p* = 0.017), mainly due to the effect of lactase usage (*p* = 0.003), but this difference in the lipid mass fraction was no longer significant on day 14 (*p* = 0.159). Among all the four treatments, the biomass produced by MC+E had the maximum lipid percentage contents on both day 8 (29.98 ± 1.92%) and day 14 (28.26 ± 9.75%). These lipid fractions were higher than those produced from cheese whey permeate (24%) [18]. On the other hand, on day 8, MG+E generated 23.85 ± 1.92% lipid content in its dried biomass, but this fraction of lipid rapidly dropped to 8.76 ± 0.86% on day 14. Such a decrease in the lipid content may be ascribed to lipid catabolism, which was observed in *Yarrowia lipolytica* strain MTYL065—another high oil-producing fungal strain [31]. Lipolytic activity has been found in the *Mucor* species, so this may cause the decrease in lipid content [32,33]. Thus, if *M. genevensis* cultivation is applied to YAW, the harvest timing of the biomass would be critical in order to obtain the greatest amount of fungal oil.

We postulate that the optimal harvest timepoint finds a balance among a legally acceptable pH (6.0–9.0) [5], low BOD, and a high net lipid mass. Based on our results, this point could be achieved between days 6 and 10 of the cultivation. This optimal timing can be further investigated along with the lipid metabolism of *M. genevensis*, for which little scientific literature currently exists.

**Figure 7:**
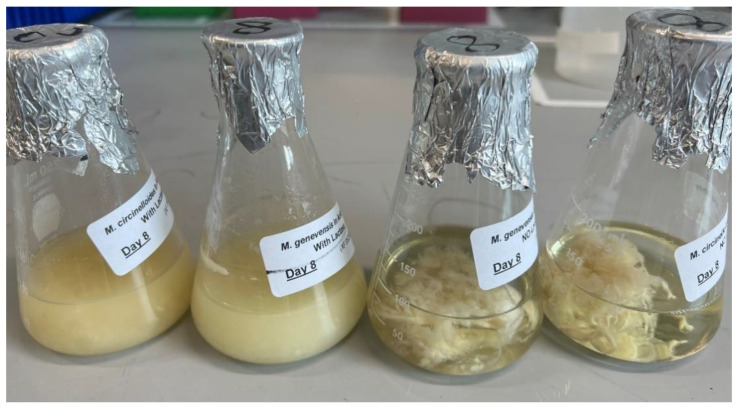
Yogurt acid whey cultures on day 8 of the aerobic cultivation of *Mucor* species at 30 °C and 200 rpm agitation. Left to right: *M. circinelloides* with lactase addition (MC+E); *M. genevensis* with lactase addition (MG+E); *M. genevensis* without lactase addition (MGNH); and *M. circinelloides* without lactase addition (MCNH). Hyphal aggregation and pelletization occurred in the latter two cultures.

Other research can potentially be conducted on the scale-up of this cultivation in a bioreactor, in which *M. circinelloides* was found to produce more biomass, a higher lipid fraction, and more γ-linolenic acid, as compared to those generated from the shake-flask method [34]. Additionally, the pelletization of *Mucor* biomass should be investigated, as this could simplify the harvesting process without compromising the yields of biomass and fungal oil [28]. In fact, aggregation and pelletization were observed in some cultures, as demonstrated in Figure 7. Such hyphal aggregates and pellets were not consistently formed throughout our experiments, but research has shown that an addition of calcium carbonate powder can facilitate pelletization [28]. Previously, Yang et al. [35] genetically engineered *M. circinelloides* and successfully increased the lipid accumulation, so experiments can also be conducted using genetically engineered *Mucor* species or other oleaginous microbes to assess their ability to produce fungal lipid in YAW. Finally, the optimization of cultivation parameters as well as the YAW substrate composition may further boost the yield of fungal lipids.

Microbial oils are seen as promising alternatives to plant oils for the production of biodiesel, which mainly consists of fatty acid methyl esters [36]. Studies have shown that high purity biodiesel can be easily produced from *M. circinelloides* biomass through a direct conversion with acid catalysts [36,37]. The biodiesel produced via such a “direct transformation” method satisfies the U.S. standards [36].

Overall, MC+E generated the largest amount of biomass and the highest lipid fraction by day 14. Nevertheless, given that our primary goal was to deacidify YAW, this treatment is not recommended as the final pH was found to be 5.37 ± 0.14, which fell below the permitted disposal pH range of 6.0 to 9.0 [5]. It is also worth noting that both *M. genevensis* and *M. circinelloides* have been isolated as contaminants in the yogurt industry [17,38], so if this treatment of YAW is applied, it should take place in a designated area separate from the yogurt manufacturing plant to avoid cross-contamination.

### 3.4. Fungal Oil Fatty Acids Composition

The fatty acid compositions of fungal oil in the *Mucor* biomasses are presented in Table 3, and the overall fatty acid profiles of both species were found to be similar to those in previous literature [18,39]. MUFAs were found to be the largest category of fatty acids among all treatments except for MGNH on day 14, in which the highest fatty acid proportion was composed of saturated fatty acids (SFAs). As the major component of MUFAs, oleic acid was the single fatty acid of the greatest proportion, ranging from 27 to 51% of the total fatty acid content in all the samples. Oleic acid is found in abundance in the Mediterranean diet, and potentially has health benefits, including the prevention of cancer, coronary heart disease, and hypertension [40]. All treatments except MC+E generated a high proportion of GLA, ranging from 7.31 to 11.4% of the total fatty acids (Table 3).

The one-way ANOVA suggests that there are significant differences among the four treatments in terms of the proportion of GLA in total fatty acids on both day 10 (*p* = 0.0274) and day 14 (*p* = 0.0491). Tukey’s test identified that MC+E has a significantly different GLA proportion from MCNH and MGNH on day 10, but it failed to identify which treatment or treatments are significantly different on day 14. The power of these tests may be limited by the number of replicates used for fatty acid analysis. GLA has been shown to have anti-inflammatory and antiproliferative potentials, and research is ongoing to explore its effects on human health [20,41]. Because of these potential benefits and given the emerging popularity of using fungal mycelia or fermentation to produce functional foods, the biomass of *Mucor* species may also be considered a feedstock for functional foods and feeds [42]. For instance, *Mucor indicus* has been studied for its use as fish feed [43], and *Mucor racemosus* has been applied in fermentations to produce functional food Douchi [44].

## 4. Conclusions

This study explored the possibility of utilizing shake-flask cultivations of *M. circinelloides* and *M. genevensis* as a treatment to deacidify YAW, while also producing biomass rich in fungal oil. Both *Mucor* species could not utilize lactose, but they could use the products of its enzymatic hydrolysis, i.e., glucose and galactose. In both treatments inoculated with *M. genevensis*, the pH increased to the allowed range for disposal (6.0–9.0) by day 6, whereas the treatment with *M. circinelloides* and lactase addition failed to reach this range during the 14-day incubation. The lactic acid concentrations also changed during the cultivations: by day 14, the treatment using *M. genevensis* with lactose hydrolysis left only 0.69 g/L lactic acid in the culture, while YAW subjected to the other three treatments still contained approximately 2.5 g/L lactic acid. The combination of lactose hydrolysis and *M. circinelloides* obtained the most biomass (0.81 g/100 mL culture) and the highest percentage of lipids (28.3% *w*/*w*) by day 14. Regarding fatty acid composition, MUFAs were found in the highest proportion on both day 10 and day 14 among all treatments except *M. genevensis* without the addition of lactase. The percentages of GLA were found to be 7.3–11.4% of total fatty acids in all treatments except for *M. circinelloides* with lactase addition, which only yielded 1.2–1.7% of GLA in total fatty acids. This research proved the feasibility of utilizing *M. genevensis* and *M. circinelloides* as a simple deacidification treatment for YAW and a potential method for the production of fungal oil that can be converted into biodiesel or even functional foods. Further research can be conducted to improve the yield of fungal oil to better compete with other oleaginous species, and to explore the industrialization of this cultivation method for the large-scale production of biodiesel.

## Figures and Tables

**Table 1:** Concentrations of lactose, galactose, and glucose (g/L) in 100 mL lactose-hydrolyzed yogurt acid whey cultures on day 0 (uninoculated), day 6, and day 14 of aerobic *Mucor* cultivations at 30 °C and 200 rpm agitation. Data are presented as mean ± standard error of the three biological replicates.

Cultures with Lactose Hydrolysis		Lactose (g/L)	Galactose (g/L)	Glucose (g/L)
Uninoculated	day 0	-- ^a^	25.48 ± 1.41	18.67 ± 0.84
*M. genevensis*	day 6	<0.04	0.04 ± 0.00	0.09 ± 0.01
	day 14	<0.04	0.03 ± 0.00	0.07 ± 0.03
*M. circinelloides*	day 6	<0.04	<0.02	0.07 ± 0.03
	day 14	<0.04	<0.02	<0.07 ^b^

^a^ Not determined due to the limitation of analysis method. ^b^ Two replicates had results of undetectable concentration of glucose (<0.03 g/L), one replicate had 0.07 g/L glucose detected.

**Table 2:** Concentrations of lactose, galactose, and glucose (g/L) in 100 mL yogurt acid whey cultures without lactase addition on day 0 (uninoculated), day 6, and day 14 of aerobic *Mucor* cultivations at 30 °C and 200 rpm agitation. Data from are presented as mean ± standard error of the three biological replicates.

Cultures without Lactose Hydrolysis		Lactose (g/L)	Galactose (g/L)	Glucose (g/L)
Uninoculated	day 0	39.11 ± 1.19	6.72 ± 0.43	0.18 ± 0.01
*M. genevensis*	day 6	40.66 ± 0.54	0.15 ± 0.01	0.14 ± 0.04
	day 14	40.96 ± 1.01	0.16 ± 0.03	<0.07 ^a^
*M. circinelloides*	day 6	39.80 ± 2.71	0.13 ± 0.00	0.10 ± 0.01
	day 14	42.77 ± 2.81	0.24 ± 0.02	<0.07 ^a^

^a^ Two replicates had results of undetectable concentration of glucose (<0.03 g/L), one replicate had 0.07 g/L glucose detected.

**Table 3:** Fatty acid composition of fungal oil obtained from the aerobically cultivated *Mucor* biomass in 620 mL yogurt acid whey. Data represent the percentage (on weight basis) of the corresponding fatty acid in the total amount of fatty acids. Data are shown as mean ± standard error for two biological replicates.

	% *w*/*w* in Total Fatty Acids							
Fatty Acid ^a^	MC+E ^b^		MCNH ^b^		MG+E ^b^		MGNH ^b^	
	Day 10	Day 14	Day 10	Day 14	Day 10	Day 14	Day 10	Day 14
Myristic acid (14:0)	3.3 ± 0.2	3.2 ± 0.3	3.0 ± 0.4	3.4 ± 0.1	1.8 ± 0.4	1.7 ± 0.0	2.4 ± 0.1	1.0 ± 0.9
Palmitic acid (16:0)	17.8 ± 0.7	17.9 ± 0.1	17.5 ± 0.2	15.5 ± 0.2	13.2 ± 2.3	15.3 ± 2.3	18.4 ± 0.7	25.6 ± 0.9
Palmitoleic acid (16:1)	9.0 ± 0.5	7.1 ± 0.3	5.5 ± 0.2	5.4 ± 0.3	11.3 ± 1.5	9.2 ± 0.1	7.1 ± 1.7	4.9 ± 0.8
Stearic acid (18:0)	10.7 ± 0.0	10.6 ± 1.0	10.7 ± 0.2	12.3 ± 0.4	7.7 ± 3.5	7.9 ± 0.5	8.5 ± 1.4	12.1 ± 0.3
Oleic acid (18:1)	49.5 ± 1.5	49.9 ± 1.1	35.7 ± 1.4	40.1 ± 1.0	40.1 ± 1.8	30.3 ± 1.6	34.8 ± 2.1	29.2 ± 1.8
Linoleic acid (18:2)	2.6 ± 0.6	4.6 ± 0.9	10.1 ± 2.4	6.8 ± 0.2	6.6 ± 0.5	9.9 ± 3.0	8.5 ± 1.4	7.2 ± 0.9
γ-linolenic acid (18:3, ω-6)	1.2 ± 0.2	1.7 ± 0.4	10.5 ± 0.7	9.2 ± 1.5	7.3 ± 0.9	10.7 ± 2.9	11.4 ± 2.8	10.7 ± 0.3
Lignoceric acid (24:0)	0.2 ± 0.1	0.1 ± 0.0	1.4 ± 0.1	1.3 ± 0.1	1.3 ± 0.1	2.0 ± 0.3	2.0 ± 0.1	2.1 ± 0.2
Total SFAs ^c^	32.8 ± 1.0	32.4 ± 2.4	34.0 ± 0.8	34.3 ± 0.0	24.2 ± 6.9	27.6 ± 2.8	32.4 ± 0.8	42.4 ± 0.4
Total MUFAs ^c^	59.2 ± 1.9	57.1 ± 0.4	41.7 ± 0.8	46.0 ± 1.7	57.1 ± 5.0	47.8 ± 2.3	43.7 ± 0.3	36.1 ± 0.8
Total PUFAs ^c^	3.64 ± 0.7	6.1 ± 1.2	19.9 ± 1.8	15.3 ± 1.6	13.5 ± 1.4	20.3 ± 5.2	19.5 ± 0.9	17.2 ± 0.6

^a^ Fatty acids that were presented in less than 1% of total fatty acids among all treatments are not listed. ^b^ MC = *M. circinelloides*; MG = *M. genevensis*; +E = with lactase addition; NH = without lactase addition. ^c^ SFAs = saturated fatty acids; MUFAs = mono-unsaturated fatty acids; PUFAs = poly-unsaturated fatty acids.

## Data Availability

The data presented in this study are available on request from the corresponding author.
